# Potential for surprising heat and drought events in wheat-producing regions of USA and China

**DOI:** 10.1038/s41612-023-00361-y

**Published:** 2023-06-02

**Authors:** Erin Coughlan de Perez, Hamsa Ganapathi, Gibbon I. T. Masukwedza, Timothy Griffin, Timo Kelder

**Affiliations:** 1 Feinstein International Center, Friedman School of Nutrition Science and Policy, Tufts University, Boston, USA; 2 Red Cross Red Crescent Climate Centre, The Hague, The Netherlands; 3 Agriculture, Food, and Environment, Friedman School of Nutrition Science and Policy, Tufts University, Boston, USA; 4 University of Sussex, Brighton, UK; 5 Zimbabwe Meteorological Services Department, Harare, Zimbabwe; 6 Climate Adaptation Services (CAS), Bussum, The Netherlands

**Keywords:** Climate-change impacts, Climate and Earth system modelling, Agriculture

## Abstract

Previous analyses of the possibility of global breadbasket failures have extrapolated risks based on historical relationships between climate and yields. However, climate change is causing unprecedented events globally, which could exceed critical thresholds and reduce yields, even if there is no historical precedent. This means that we are likely underestimating climate risks to our food system. In the case of wheat, parts of the USA and China show little historical relationship between yields and temperature, but extreme temperatures are now possible that exceed critical physiological thresholds in wheat plants. UNprecedented Simulated Extreme ENsemble (UNSEEN) approaches use large ensembles to generate plausible unprecedented events, which can inform our assessment of the risk to crops. We use the UNSEEN approach with a large ensemble of archived seasonal forecasts to generate thousands of plausible events over the last 40 years and compare the results with historically observed extreme temperature and precipitation. In the US midwest, extreme temperatures that would have happened approximately 1-in-100-years in 1981 now have a return period of 1-in-6 years, while in China, the current return period is on the order of 1-in-16 years. This means that in the US midwest, extreme temperatures that used to have a 1% chance to occur in 1981 now have a 17% chance to occur in any given year, while in China, the chance increased from 1% to 6%. Record-breaking years exceeding critical thresholds for enzymes in the wheat plant are now more likely than in the past, and these record-breaking hot years are associated with extremely dry conditions in both locations. Using geopotential height and wind anomalies from the UNSEEN ensemble, we demonstrate that strong winds over land pull dry air towards the regions these during extremely hot and dry unseen events. We characterize plausible extremes from the UNSEEN ensemble that can be used to help imagine otherwise unforeseen events, including a compound event in which high impacts co-occur in both regions, informing adaptation planning in these regions. Recent temperature extremes, especially in the US midwest, are unlikely to be a good proxy for what to expect in the next few years of today’s climate, and local stakeholders might perceive their risk to be lower than it really is. We find that there is a high potential for surprise in these regions if people base risk analyses solely on historical datasets.

## Introduction

Given the global interconnectedness of the world’s food system, simultaneous shocks to major food grain production areas (breadbaskets) can dramatically influence the price and availability of staple foods. Several studies have attempted to quantify the risk of multiple breadbasket failures due to climate shocks alone^1–3^. These studies have primarily extrapolated from historical patterns, quantifying the risk that climate shocks from the past could happen simultaneously in the future. However, climate change brings new and unprecedented events that can have consequences different from those experienced in the past, and history-based analyses might therefore under-estimate our current risk. In this study we depart from a focus on historical events, instead demonstrating how to visualize the risk of historically unprecedented events that might cross critical thresholds in major wheat-producing regions of the USA and China.

Most studies quantifying the risk of crop failure use historical relationships between climate and crop yields as the basis for assessing how future or unprecedented climate states might affect yields. For example^2^ use historical yields to define a threshold for severe water stress in maize-growing regions of the US and China, and then they examine the change in risk of this threshold using large ensembles to simulate unprecedented extremes. Estimates of the risk of multiple breadbasket failures for different crops also take this approach, first estimating climate-yield relationships from historical data, and then extrapolating yield results based on changes to temperature and precipitation variables that were historically related to yield^4^ In some regions, more than 50% of historical yield variability can be attributed to weather^5^.

However, in a changing climate, climate-yield relationships will change. Never-before-experienced climate states and unprecedented events can have greater effects on crops than might be expected from a simple extrapolation of historical association. In particular for temperature, we might expect that never-before-experienced high temperatures could cause crop loss, even if there is no historical relationship between yield and temperature. Non-linearities in the response of crops to heat stress can mean the future looks distinctly different from the past. In addition, climate stressors can combine with other pressures to threaten agricultural productivity; these include conflict, pests, disease, soil health, seed quality, and irrigation, for example.

Wheat (*Triticum aestivum* L.) yields in parts of the United States and China do not show a strong relationship with temperature in observed or simulated datasets for the past^6^, and therefore extreme temperatures in these regions are not often included in models of potential breadbasket failure^4^. However, physiological models demonstrate that wheat plants are sensitive to temperature in several critical growth phases^7^. Generally, prolonged periods of extreme heat result in accelerated leaf senescence and a reduction in leaf expansion and radiation use efficiency. Short duration heat events are particularly harmful during sensitive development phases such as stem elongation. Heat extremes during grain filling can cause a reduction in the growth rate and the grain number^8,9^, while heat stress during anthesis and may result in partial or complete sterility of the florets^10,11^.

Simulations for the end of the century show that unprecedented temperatures are likely to affect yields as higher thresholds are crossed^12^ In fact, process-based and statistical models tend to agree that warming should negatively impact wheat yields^13,8^, and a review of different model types found agreement that global wheat yield is likely to be negatively impacted by increasing temperatures with climate change^14,15^. One solution to assess the impact of this nonlinearity is to use crop model simulations that can incorporate critical thresholds^16,17^ However, many of these crop models are developed based on historical yields, and many of them focus on annual extremes and “likely” ranges, rather than low-likelihood high-impact events.

New methods to simulate unprecedented extremes can expand our understanding of what is possible, beyond historical events. Large ensembles of physics-based climate models can provide a larger sample of “alternative realities” to calculate extreme value statistics^18–20^ One example is the UNprecedented Simulated Extremes using ENsembles (UNSEEN) approach, using large ensembles of archived forecasts to better understand extremes^21^.

To date, most studies of UNSEEN events or climate storylines have departed from a historical extreme event that has already happened, assessing plausible changes in frequency and magnitude (e.g. storm Desmond^22^). The approach has also been used to derive future impact analogs of historical events, such as a soybean (*Glycine max* (L.) Merr.) drought in the future^17^.

The UNSEEN approach can also be used to explore synthetic events—events with no historical analog—if the models have been properly assessed for their ability to produce realistic events^23^. Climate storylines that illustrate how record-breaking extremes might occur can expand our imagination to capture events that are plausible, yet never before experienced. Given that adaptation to climate change tends to be prompted by people’s lived experience of extreme events^24–27^ visualizing such events before they happen can support preparedness and climate change adaptation.

In this study, we use the UNSEEN approach to examine storylines of unprecedented heat in two wheat-producing regions of the world’s breadbaskets, the USA and China. First, we assemble a large ensemble of archived forecasts for each region for temperature and precipitation, estimating the frequency of temperatures above critical growing thresholds. We estimate changes to the return periods of extreme temperatures with climate change, and consider the probability of a compound extreme of high temperatures and low rainfall in each region. While many other studies have focused on climate change in the far future, we explore the current-day climate, and how risks have already changed from the recent past, complementing work^1^.

## Results

### UNSEEN evaluation in Midwestern USA and Northeastern China

**Fig. 1:**
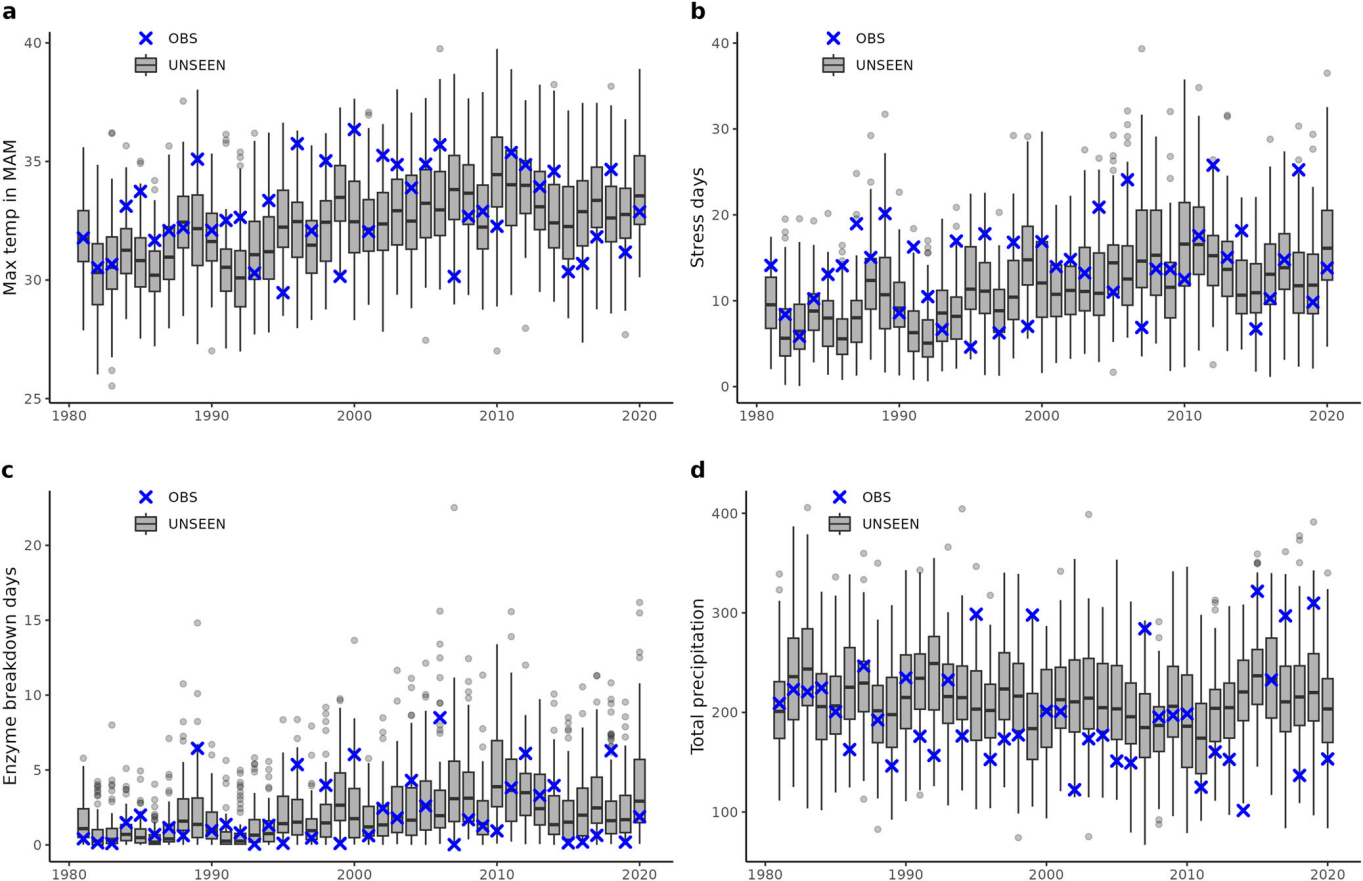
UNSEEN events in the US study region. Historical observations of temperature and precipitation in March–May in USA Midwest winter wheat producing region (blue crosses), overlaid on gray boxplots of the UNSEEN large ensemble. Boxplots visualize the illustrated as the median, interquartile range, 1.5x interquartile range and outliers. Plots are for the following variables: (**a**) Maximum temperature, (**b**) Number of days above the “stress” threshold of 27.8 °C, **c** number of days above the “enzyme breakdown” threshold of 32.8 °C, and (**d**) Total precipitation.

In the US midwest, the UNSEEN ensemble shows a steady rise in maximum temperatures that are possible over time; the interquartile range of the ensemble results used to fall below 30 °C, and now the upper end of the interquartile range approaches 35 °C (Fig. 1). Historically, the maximum temperatures recorded for the past 40 years have been lower than the extremes produced by the UNSEEN ensemble. The highest values in the large ensemble for recent years reach 40 °C, while the highest values in the observational dataset are around 37 °C.

The number of days that exceed critical heat thresholds have also been increasing in both the observed and modeled datasets for the US midwest. The UNSEEN ensemble contains discrete events that are well beyond the observed record, including one event with more than 20 days exceeding the “enzyme breakdown” threshold.

There is no clear trend in rainfall in the observed or simulated datasets of March–May in the US midwest (Fig. 1d). The 2014 historical drought is close to the most extremely dry events simulated in the UNSEEN dataset, although there are a few UNSEEN events that are drier than this historical record. Such events could negatively impact wheat yields, as happened in 2014. In Kansas in 2014, the wheat monitor reported that “wheat condition declined all month and, by the end of May, 62% of the crop was reported to be in very poor to poor condition, compared to 47% at the beginning of the month and 45% last year”^28^ The yield per harvested acre was the lowest since 1995^28^ News reports from local public radio explained that “persistent drought, harsh winds and below normal winter temperatures, combined with already low sub-soil moisture levels, have decimated the winter wheat crop in Kansas, Oklahoma, and Texas. These States make up the heart of the wheat belt—even with drought-affected low yields last year, they still produced one-third of the national winter wheat crop”^29^.

**Fig. 2:**
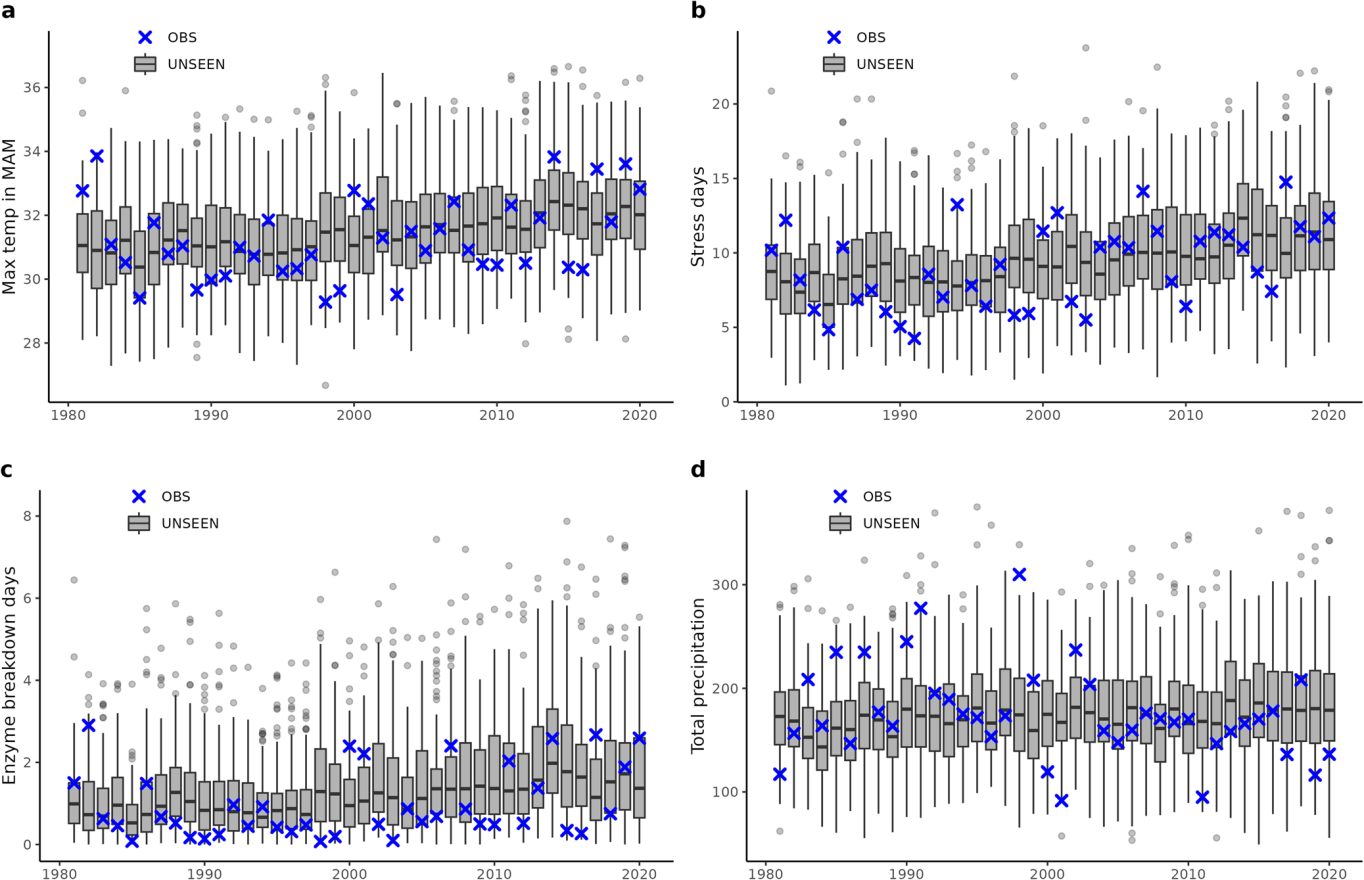
As in Fig. 1, for the China winter wheat region. Historical observations of temperature and precipitation in March–May (blue crosses), overlaid on gray boxplots of the UNSEEN large ensemble. Boxplots visualize the illustrated as the median, interquartile range, 1.5x interquartile range and outliers. Plots are for the following variables: (**a**) maximum temperature, (**b**) number of days above the “stress” threshold of 27.8 °C, (**c**) number of days above the “enzyme breakdown” threshold of 32.8 °C, and (**d**) total precipitation. Note that plot (b) did not pass the fidelity test and therefore should be interpreted with caution, as the kurtosis of the observed data is outside the 95th percentile of the UNSEEN ensemble.

In China, results are similar (Fig. 2). Maximum temperatures in March–May show an increase with time, and the large ensemble includes many unprecedented events. This includes temperatures in the high 30 s, while the historical record is closer to 35 °C. The number of “stress” days and “enzyme breakdown” days are both increasing, with UNSEEN possibilities of more than 10 days in which the “enzyme breakdown” threshold is exceeded in one season.

The UNSEEN ensemble also contains several record-breaking drought events that have lower rainfall than ever observed in the region. These are physically plausible events that are drier than what has been historically observed. Hotter and drier summers can improve sowing and harvesting conditions and reduce the risk of waterlogging. However, should these periods coincide with sensitive crop development phases e.g. flowering and grain filling, this can result in relatively lower yield outcomes.

### Increasing probability of extremes

**Fig. 3:**
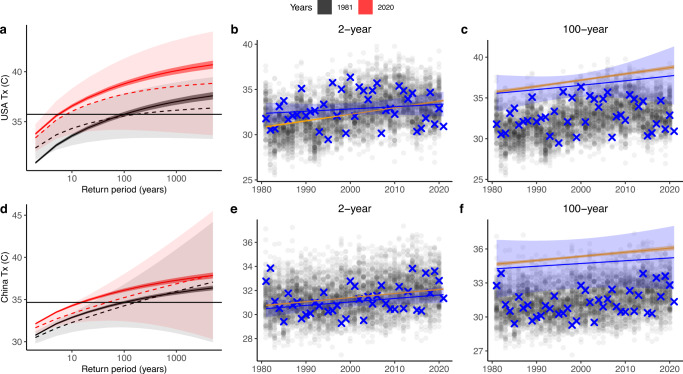
Extreme value distributions for daily maximum temperatures. Temperatures are in March–May for the US midwest (top row) and northeast China (bottom row). **a**, **d** Return period of seasonal maximum temperatures in 1981 and 2020. GEV fits are plotted for observations using dotted lines and light shading to indicate the uncertainty estimates. GEV fits are plotted for the UNSEEN ensemble using solid lines and dark shading for uncertainty estimates. All GEV fits are non-stationary distributions with covariates for the years 1981 and 2020. The magnitude of the 100-year event is indicated with a black horizontal line. **b**, **e** UNSEEN ensemble in gray overlaid with observations in blue, with a non-stationary 2-year return period estimation for each dataset. **c**, **f** UNSEEN ensemble in gray overlaid with observations in blue, with a non-stationary 100-year return period estimation for each dataset. The 95th percentile confidence interval is plotted using shading for the observations in blue. Statistical uncertainty is estimated as 95% confidence intervals based on the normal approximation.

Over time, the UNSEEN ensemble demonstrates a discernible change in the likelihood of extremely hot temperatures in both USA and China regions. Figure 3 plots the extreme value distribution fitted to the observations and the UNSEEN ensemble for maximum temperature in the March–May season. In both cases, maximum temperatures are higher now than in the 1980s, with a 1-in-100 year event in 1981 happening on average more often than every 6 years in 2020 in the US midwest. The simulated change is slightly less in northeast China, with a 1-in-100-year event in 1981 happening on average approximately every 16 years in 2020 (Fig. 3a, d). This translates into a 1% chance of the event happening in 1981, moving to a 17% (USA) and 6% (China) chance of happening in the year 2020.

In both case study locations, however, the best-fitting extreme value distribution for the observational dataset is a stationary GEV fit. This is in contrast to a non-stationary fit for the UNSEEN ensemble. If one were to simply extrapolate a nonstationary GEV fit from observational data in the USA study region (Fig. 3a, dotted line), for example, one would estimate lower values and much larger uncertainties as compared to the dynamically consistent UNSEEN ensemble, thus representing the strength of this type of analysis.

Assuming that the model is accurately representing the range of today’s climate, this could indicate that both regions have been “lucky” in recent years, and both regions have not experienced the full range of high temperatures that are now possible in today’s climate. In fact, these regions have been selected for wheat production partially because of favorable climate conditions in the past, and the critical thresholds were essentially boundaries. That is no longer the case, and extreme temperatures are much more likely. Recent memory of temperature extremes is on the lower end of the distribution of plausible extremes for today’s climate, especially in the US midwest where the difference between the observed and UNSEEN trend is highest. According to the UNSEEN ensemble, an event that would have been a 1-in-100 year maximum temperature event for March–May in the US midwest in 1981 is now a 1-in-6 year event. Other studies have similarly detected long term positive trends in temperature in both regions, with some attribution to anthropogenic climate change^30–34^. Whereas attribution studies compare the current climate with a pre-industrial climate, here, we are able to discern trends in the recent decades which might be of value in referencing people’s recent lived experiences.

### Record-breaking extreme heat and drought

As expected given the stochastic nature of weather, we find that historical weather observations are limited in their range compared to a large ensemble of plausible weather outcomes. The UNSEEN ensemble contains a variety of heat and drought events for each location that would break historical records.

**Fig. 4:**
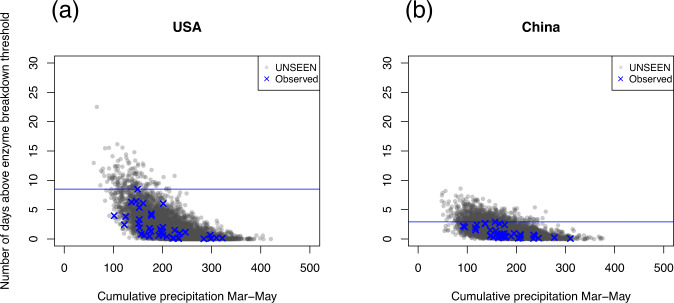
Extreme heat vs extreme dryness. Cumulative precipitation plotted against the number of days crossing the “enzyme breakdown” threshold in March–May for (**a**) USA and (**b**) China. UNSEEN ensemble members are plotted in gray, overlaid with observations in blue. The historical record for number of “enzyme breakdown” days is plotted as a blue horizontal line; any gray UNSEEN events above that line are record-breaking.

Extreme heat and extreme dryness are not independent of each other, often occurring simultaneously as a result of blocking weather patterns. Therefore, we plot the relationship between extreme heat and dryness in each location in Fig. 4. In both regions, extreme heat is strongly associated with dryness, and very wet events do not co-occur with extreme heat.

If there is a record-breaking hot season in which the number of days above the enzyme breakdown threshold is higher than experienced in the past (higher than the blue line in Fig. 4), it is likely to also be a dry season. In the US midwest, the UNSEEN ensemble produces 161 record-breaking seasons with high temperatures, and while most of them are relatively dry, 14% of them have extremely low rainfall that is less than the worst drought on record, the drought of 2014. This also applies in the other direction, of the 31 UNSEEN drought events that are worse than the worst drought experienced in the last 40 years, 71% of these events also have record-breaking heat.

In China, the results are similar; 63% of the UNSEEN record-breaking drought events are also record-breaking heat events in terms of the frequency of days above the enzyme breakdown threshold. We can imagine event-based storylines of extreme heat and extremely low rainfall that would cause unprecedented impacts at the intersection of these two hazards. Higher temperatures also produce higher evaporation rates, which can further reduce water availability for agriculture, beyond the record-breaking low precipitation.

### Individual extreme events

**Fig. 5:**
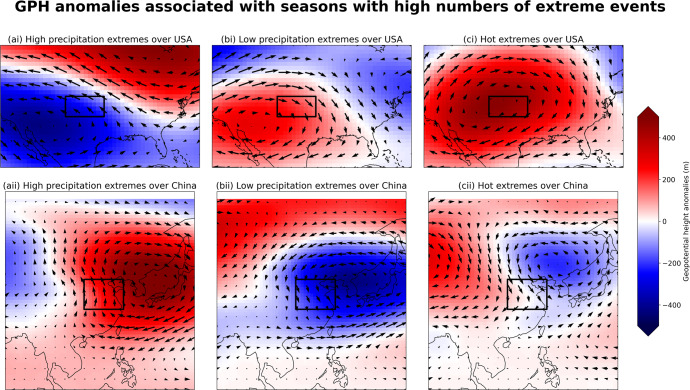
Pressure and wind anomalies for UNSEEN events. Composites of geopotential height and wind anomalies at 500 mb associated with the most extreme events in the UNSEEN ensemble for the study region. Each plot is a composite of the 10 seasons of (**a**) highest precipitation, (**b**) lowest precipitation, and (**c**) highest number of enzyme breakdown days in each study area. The first row depicts the USA study area, and the second row the China study area, both delineated with a black box. Individual plots for each of the 10 events used to make these composites are available in the Supplementary Information.

One of the primary advantages of analyzing a large ensemble of physically plausible events is that the ensemble allows users to examine the drivers and physical contributing factors for specific extremes that have never happened in the observed record. In Fig. 5, we plot composites of geopotential height (GPH) anomalies and wind anomalies at the 500 mb pressure level, to analyze the most extreme events from the larger ensemble.

In the USA region, the 10 driest March–May seasons in the SEAS5 ensemble (Fig. 5bi) are dominated by northerly and westerly wind anomalies. Such wind anomalies pull dry air from the continental USA to the study region, limiting precipitation. These are produced by positive seasonal anomalies of geopotential height to the west and south of the study region. In most of these 10 UNSEEN events, the positive anomalies are concentrated in the southwest of the USA, but there are individual events where the positive anomalies extend more widely across the USA (see Fig. SI11 for plots of anomalies associated with individual UNSEEN events).

Wind anomaly patterns are similar for the hottest seasons (Fig. 5ci); seasons with the largest number of hot days above the enzyme breakdown threshold are characterized by large regions of high pressure over the study area and to the southwest (Fig. 5ci). There are likely land-atmosphere feedbacks that can strengthen the heating effects during anomalously dry events^35^. In contrast, the wettest events (Fig. 5ai) have seasonal wind anomalies from the south and east, bringing moisture from the Gulf of Mexico and Atlantic (Fig. SI11).

In the China study region, wind anomalies are also critical to generating the extremely wet, dry, and hot events in Fig. 5, second row. The driest seasons (Fig. 5bii) modeled in the SEAS5 ensemble had wind anomalies from the north and west, bringing air over land towards the study area. This was associated with a low-pressure zone to the northeast of the study area. Hot seasons with the highest number of days above the enzyme breakdown threshold (Fig. 5cii) had similar wind anomalies as the low-precipitation events, and they also show a low pressure region to the northeast of the study area.

In eastern China, the 10 wettest seasons have wind anomalies from the opposite direction, coming from the south and east, bringing moisture to the study region (Fig. 5aii). These very wet seasons are associated with strong high-pressure regions to the northeast of the study area, generating clockwise winds that pull moisture from over the oceans to the winter wheat region. In July 2021, there was an extreme rainfall event in Henan province, once of the regions within our study area, and subsequent meteorological analyses identified that this was indeed caused by winds coming from the east, bringing moisture to the region^36^ similar to the synthetic events pictured in Fig. 5a.

In both regions, wind anomalies coming from land are associated with hot/dry seasons, and the opposite direction of wind anomalies over water are associated with extremely wet seasons, as might be expected. However, the patterns of geopotential height anomalies that produce such wind anomalies have some variety in their general shape, size, and location. Atmospheric anomalies associated with individual UNSEEN events are plotted in Supplementary Figs. 10–15. For example, while northwesterly wind anomalies in the China winter wheat region are associated with a low-pressure zone to the northeast, this zone is larger in some realizations (e.g. Fig. SI 14B), and extends further south in other realizations (e.g. Fig. SI 14A and SI 14F). Therefore, meteorologists and climate scientists can be alert to several different varieties of the same pattern, which can all produce the wind anomalies that are associated with extremely hot/dry conditions in the region that produces winter wheat.

### Compound events

The UNSEEN approach can be used to detect whether the likelihood of simultaneous extremes in both regions is higher than would be expected from random chance. In the observational datasets, there are no correlations between the two study regions of USA and China for maximum temperatures or total precipitation in the March–May season. In the UNSEEN ensemble, total precipitation remains uncorrelated between the two regions, but there is a small correlation for maximum daily temperature. The TXx correlation is 0.06 with 95% confidence intervals of 0.03-0.09. This is likely due to the influence of climate change on extreme temperatures globally, which affects both regions. See Supplementary Fig. 9 for a scatterplot of the temperatures.

**Fig. 6:**
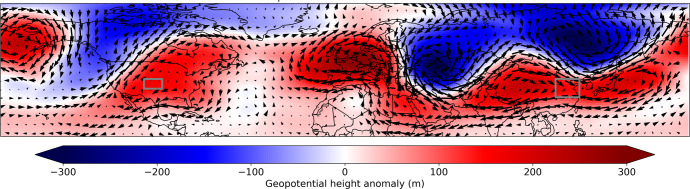
Pressure and wind anomalies for compound events. Composites of geopotential height and wind anomalies at 500 mb associated with concurrent extreme events in the UNSEEN ensemble in both study regions. Each plot is a composite of the 10 seasons that produced an extreme number of enzyme breakdown days in both study areas. The two study areas are delineated by gray boxes. Individual plots for each of the 10 events used to make these composites are available in Supplementary Fig. 16.

While there is not a strong relationship between the two regions, there are individual UNSEEN events that do happen to produce simultaneous extremes in both locations. We identified the top 250 ensemble members for each study region that produced the greatest numbers of enzyme breakdown days, and there are 10 ensemble members that overlap in those two lists, producing extreme heat simultaneously in both locations. Figure 6 illustrates a composite of the geopotential height and wind anomalies associated with these 10 events, which are extreme in both study regions. This represents a dynamically consistent event-based storyline of a co-occuring event in both locations. The composite seems to be associated with a zonal wavenumber-3 disturbance in the higher latitude circulation, creating high pressure systems over both study areas. This compound event simultaneously creates the conditions seen in Fig. 5ci (USA) and 5cii (China) (Table 1).

**Table 1:** 100-year return periods for the daily maximum temperature in March–May, for 1981 and 2020 in both study regions, as simulated by the UNSEEN ensemble.

Year	USA	China
1981	35.7 °C	34.7 °C
2020	38.7 °C	36.1 °C

Plots of individual compound events are available in Supplementary Fig. 16. The ensemble that generated the most extreme compound event was an UNSEEN ensemble member in 2018 (Fig. SI 16J), which shows similar atmospheric patterns to this composite. It simulated an event that had an regional average of 12.9 enzyme breakdown days in the USA study region (the observed record is 8.5), and in China, this event produced a regional average of 5.2 enzyme-breakdown days (observed record of 2.9).

## Discussion

Climate change presents a key risk to food systems globally, but many risk analyses derive estimates based on past climate-yield relationships, without accounting for the fact that we live in a fundamentally changed climate. Nonlinearities in the response of crops to our changing climate can present unexpected consequences in terms of failed crops and reduced yields. Novel techniques to create UNSEEN ensembles of plausible alternative seasons can expand our imagination of what types of never-before-experienced events are now possible, and this can enable modeling and stimulate discussion of what kind of impacts these could have on agriculture.

In the case of non-irrigated winter wheat in the USA and China, we demonstrate that several regions might have been “lucky” in terms of their recent experience of extreme events. Given the stochastic nature of weather, recent temperature extremes in the US midwest have happened to be cooler than the full range as simulated by the UNSEEN climate ensemble. This means that recent years are unlikely to be a good proxy for what to expect in the next few years of today’s climate, and local stakeholders might perceive their risk to be lower than it really is. Previous studies estimating the vulnerability of global breadbaskets to climate extremes could contribute to this lower perception of risk, because they failed to take temperature into account due to historical (lower) temperatures not affecting wheat yields in these regions.

In the large ensemble analyzed here, we find that extremely hot and dry seasons are associated with large-scale circulation anomalies, with winds bringing dry air over land to both wheat-growing regions. While synoptic-scale heatwave events might be associated with localized regions of high pressure, we find that the most extreme seasons have large-scale circulation anomalies, often forming gradients of pressure with strong wind anomalies. These large scale circulation anomalies can be monitored to better understand and forecast the conditions that are likely to cause stress for wheat crops in each region.

An UNSEEN approach can allow us to imagine some of these unprecedented climate events that can interact with other drivers of crop supply globally, telling the story of what could happen in two regions of the world’s breadbaskets that have so far been “lucky”. The approach is constrained by the ability of models to represent the full range of plausible outcomes in a location, and while we included fidelity/stability/independence tests on the data, our models might not be fully representing the spectrum of risk^23^ New methodologies to perturb models and simulate plausible extremes can support these explorations of climate storylines and unprecedented events to enable adaptation^20^.

Given the substantial possibility of record-breaking heat, likely in combination with drought, in both the winter wheat producing regions of the USA and China, climate change adaptations for heat and drought will be needed in many of the world’s breadbaskets. Changes to agricultural management are widespread for most staple grains, including wheat. Adaptations include research to find genetic improvements to wheat varieties that preserve yields in drier and hotter conditions^37,38^. Higher-yielding wheat varieties have historically been more sensitive to hot temperatures above 34 degrees^39^, therefore further research into hybrid cultivars that can withstand extreme heat will be important. Agriculturalists have also experimented with changing planting dates and changing maturity dates of crops^40^ and movement of agricultural zones^41^. Strategies for managing drought risk include irrigation opportunities and agricultural management to store additional soil water that can be used by the crop.

Adaptation investments tend to be spurred by personal experience of extreme events. Results from UNSEEN ensembles such as this one can be used to generate storylines of record-breaking climate events, which can help people visualize impacts without needing to experience them directly. Further investment in the UNSEEN and storyline approach has the potential to reveal gaps in our perception of current risks to our breadbaskets and food systems, encouraging adaptive action to prevent negative impacts in the coming years.

Climate shocks do not act in isolation, and they interact with a variety of other pressures on agricultural production. These include domestic policies, pests and disease, global trade, planting areas, irrigation, and others. For example, at the time of writing, the 2022 war in Ukraine has reduced the supply of wheat from the breadbaskets in Russia and Ukraine^42^ Pressures have included damage or blocks to export infrastructure, sanctions, and shifts in regional control^43^ Of all these agricultural pressures, we understand climate events well through physics-based models. Therefore, the use of such models to encourage planning for extreme events can help reduce climate pressures on agriculture in the future.

## Methods

### Study design

We follow the protocol to apply and ensure credibility of UNSEEN^23^ First, the domains, variables, and indices are defined that are most relevant to wheat growth. We then select appropriate datasets for the analysis, and statistically evaluate the realism for the selected event definitions. When issues are identified, these are mentioned and/or resolved through reducing the sample size or correcting the data. Statistics of interest are then obtained from the datasets that are deemed realistic.

### Variables

In this study, we focus on winter wheat in the USA and China, specifically each country’s main production region. In the US, this is the midwest of the USA, including western Kansas (27% of national production), eastern Colorado, and northwestern Oklahoma (105–95.5 W, 35–40 N)^44^. The China region is northeastern China, including Hebei, Shandong, Henan, Jiangsu, and Anhui provinces (110–122.5E, 30.5–40 N), each of which account for 10% or more of China’s winter wheat production^45^ In the USA, the states of Kansas, Oklahoma, and Colorado collectively produced 15,229,953 metric tons in 2017, which was 43.5% of the country’s winter wheat crop^46^ In total, China produced 134,334,000 metric tons of wheat in 2017, including winter wheat and other varieties. Total global wheat production in 2017 was 772 million metric tonnes (FAO).

Winter wheat growth begins in autumn, followed by a dormancy period through the winter^47^ Regrowth starts in early spring, with full grain flowering and development occurring in the early to late spring, making this period critical for yield growth before harvest in June and July^47^. In previous studies, wheat yield in each location was correlated with cumulative precipitation during the growing season, with no or little relationship to temperature^3^^,6^.

However, temperatures above 27.8 °C in May have been associated with heat stress on winter wheat plants^47^ Prolonged durations at or above this temperature are particularly detrimental. Additionally, above temperatures of 32.8 °C, wheat enzymes start to break down, further damaging development of the plant^48^ Other studies have used thresholds of 21 °C as optimal, and 34 °C as damaging, to demonstrate some reduction in yield due to extremely hot temperatures in parts of Kansas^49,39^. Given that harvest happens in June and July, the stages of wheat that are sensitive to heat stress (including anthesis and grain filling) tend to happen between March and May. While there is some irrigation in both regions, winter wheat relies on rainfall for much of its water requirements, and therefore, total precipitation is critical during March–May as well.

In this study, we selected three temperature values of interest: the maximum daily temperature in the March–May season, as well as the number of days with a maximum temperature above 27.8 °C (stress threshold) and number of days above 32.8 °C (enzyme breakdown threshold). We also analyze the total precipitation in March–May as a critical influence on wheat outcomes. Each variable is calculated at the native resolution of the dataset and then area-averaged over land points in the selected region.

### Datasets

For the large ensemble of “alternative realities” of the current climate and recent past, we use archives of SEAS5, the long-range forecasts from the European Centre for Medium Range Weather Forecasting (ECMWF)^50^ These forecasts are initialized on the first of every month and run a physics-based model for 7 months into the future, generating daily weather data for a 7-month time period. Forecasts that include the full March–May season are those that are initialized in March (1 month of lead time), February (2 months of lead time), January (3 months of lead time), December (4 months of lead time), and November (5 months of lead time).

While SEAS5 is run as an operational forecast, the archived large ensemble can be used to identify extreme events that have not been experienced before, because the archived forecasts contain plausible events that simply never happened. The model consists of 25 ensemble members from 1981-2016, and from 2017 to the present contains 51 ensemble members. Therefore, including each of the 5 lead times, the large ensemble contains 125 alternative realizations of each year until 2016, and 255 ensemble runs per year from 2017 onwards.

To verify the SEAS5 ensemble, we compare results against historical observations. In the USA, we use the DayMet dataset version 4, a 1 km by 1 km daily surface weather dataset for North America derived primarily from ground-based weather stations^51^ (In China, we use the ERA5 Land reanalysis of daily surface weather, which is produced at 9 km resolution^52^. All datasets were scaled to 1 degree resolution for the purpose of the analysis.

### Evaluation of UNSEEN

For each of the four variables of interest, we evaluate the SEAS5 UNSEEN ensemble to determine which ensembles and lead times could be used to characterize the full range of plausible events. First, we evaluated the stability across lead times, to measure whether there was any model drift in the longer lead-times. None was found for any of the variables and locations in this study (plots available in Supplementary Figs. 3, 4).

We then estimated the independence between ensemble members. Because all ensemble members were initialized at the same moment, there is often a lack of independence between ensemble members at shorter lead-times. The first lead time (a forecast initialized on March 1 for the March–May season) was excluded for all variables due to assumed interdependence of ensemble members. Beyond that first lead time, we eliminated any lead-times for which pairwise rank correlations between ensemble members had a median value greater than 0.25, demonstrating a lack of independence^53^ This only occurred once, for TXx in the USA region, for which lead 2 was removed (see Figs. SI 1-2 for plots of the independence between lead times).

Lastly, we estimated the fidelity of the SEAS5 UNSEEN ensemble in comparison to the historical observational dataset for TXx and for Total Precipitation. We randomly subsample the larger UNSEEN sample into 10,000 series of the same length as the observations, to create “proxy observations” to compare with the observed data. We compared the mean, standard deviation, skewness, and kurtosis of the observed dataset against those values for each of the simulated observations. In the case of Total Precipitation in the China region for MAM, the mean of the historical observations fell outside the 95th percentile range of the UNSEEN ensemble, and we implemented an additive bias-correction. The UNSEEN total precipitation in March–May was adjusted by subtracting 24 mm to match the mean of the observations. The other variables were not adjusted (see Supplementary Figs. 5–8 for the fidelity plots for all variables). However, in the case of TXx for the USA region, the observed kurtosis was slightly below the 95th percentile of all kurtosis results from the UNSEEN ensemble. Results for this variable should therefore be interpreted with caution.

For the derived extremes variables of number of days above the stress/enzyme breakdown thresholds, we carry out the same fidelity tests. The standard deviation, skewness, and kurtosis of the historical observations were within the 95th percentile range of the UNSEEN results, with one exception. The observed number of days above the “stress” threshold in the China region is below the 5th percentile of the UNSEEN ensemble, and therefore should also be interpreted with caution.

While the UNSEEN approach might not capture the full range of all plausible events, these fidelity checks allow us to proceed with some confidence that the simulated extremes are worth exploring to inform adaptation planning.

### Statistical analyses

Using these validated datasets, we then obtained insights into low-likelihood high-impact events using three approaches. First we visually inspected the time series of observed and UNSEEN events. Given that the UNSEEN dataset contains a large number of events per year, this dataset is represented using boxplot statistics, showing the median, interquartile range, 1.5 x interquartile range, and members outside the 1.5 x interquartile range. Secondly, we count the number of threshold exceedances. The probability of such threshold exceedances can be expressed as the percentage of the number of exceedances to the total number of events. As such, the likelihood of compound events can also be estimated. Thirdly, we applied extreme value statistics to March–May maximum temperatures^54^ We fitted a Gumbel, Generalized Extreme Value (GEV), and non-stationary GEV distribution to the historical results and the UNSEEN ensemble. We fitted the location and scale parameters linearly to time as a covariate^19,23^, as in Kelder et alWe test which distribution best fits the data using a likelihood-ratio test and estimate the parameters of the distributions using maximum likelihood estimation (MLE). These distributions were used for the calculation of the likelihood and magnitude of exceptionally extreme temperatures in the historical and current climate. To analyze circulation patterns related to the UNSEEN extreme events, we plot the geopotential height (GPH) anomalies and wind anomalies at the 500 mb pressure level, to analyze specific extreme events from the larger ensemble. Using leads 1–4 (due to availability of monthly SEAS5 data), we plot the 10 driest, wettest, and hottest seasons in the USA and China study regions.

### Supplementary information

Supplementary Information Coughlan de Perez et al. Wheat UNSEEN USA China

## Data Availability

All data used in this study is publicly available, and can be accessed as follows: SEAS5 Archives and ERA5 Land on the Copernicus Climate Data Store: https://cds.climate.copernicus.eu/cdsapp#!/dataset/seasonal-original-single-levels?tab=form. 10.24381/cds.181d637e. https://cds.climate.copernicus.eu/cdsapp#!/dataset/reanalysis-era5-land?tab=form. 10.24381/cds.e2161bac. Daymet data on the ONRL DAAC website: https://daymet.ornl.gov/getdata. 10.3334/ORNLDAAC/2129.

## Code availability

The UNSEEN methodology is documented here: https://unseen-open.readthedocs.io/en/latest/. Any requests for code or data can be directed to the authors.
