# Supplementary material for: Potential for surprising heat and drought events in wheat-producing regions of USA and China
Source: NPJ Clim Atmos Sci. 2023 Jun 2;6(1):56. doi: 10.1038/s41612-023-00361-y (PMC11041665; doi:10.1038/s41612-023-00361-y)

Supplementary Information

Coughlan de Perez, E., Ganapathi, H., Masukwedza, G. I., Griffin, T., & Kelder, T.. *High potential for surprising heat and drought events in wheat-producing regions of USA and China*.

Supplementary Figure 1. Independence across lead times, showing pairwise rank correlations between ensemble members for TXx, maximum temperature in the Mar-May season. 2

Supplementary Figure 2. As in SI1 for total precipitation in Mar-May. No leadtimes were excluded. 2

Supplementary Figure 3. Stability across lead times for TXx, showing the climate variable plotted by lead time, to verify that none of the lead times show drift. (A) USA region, (B) China region 3

Supplementary Figure 4. As in SI2 for total precipitation in Mar-May. 3

Supplementary Figure 5. Fidelity of the ensemble to observations for TXx in USA region in terms of the mean, standard deviation, skewness, and kurtosis. 4

Supplementary Figure 6. As in SI5 for total precipitation, USA region in Mar-May. 5

Supplementary Figure 7. As in SI5 for TXx in Mar-May, China region. 5

Supplementary Figure 8. As in SI5 for total precipitation in Mar-May, China region. 6

Supplementary Figure 9. Relationship between temperatures in northeastern China and midwest USA study regions in the Mar-May season. 7

Supplementary Figure 10. Geopotential height and wind anomalies at 500mb associated with the 10 wettest March-May seasons in the USA study area. As in Figure 5 in the main text. 8

Supplementary Figure 11. As in SI10; geopotential height and wind anomalies at 500mb associated with the 10 driest seasons in the USA study area. 8

Supplementary Figure 12. As in SI10; geopotential height and wind anomalies at 500mb associated with the 10 seasons of most days above enzyme breakdown threshold in the USA study area. 8

Supplementary Figure 13. As in SI10; geopotential height and wind anomalies at 500mb associated with the 10 wettest March-May seasons in the China study area. 9

Supplementary Figure 14. As in SI10; geopotential height and wind anomalies at 500mb associated with the 10 driest seasons in the China study area. 9

Supplementary Figure 15. As in SI10; geopotential height and wind anomalies at 500mb associated with the 10 seasons of most days above enzyme breakdown threshold in the China study area. 9

Supplementary Figure 16: Compound events. As in SI10, geopotential height and wind anomalies at 500mb associated with the 10 events that produce the most days above enzyme breakdown threshold in BOTH the USA and China study regions. 10

Supplementary Figure 1. Independence across lead times, showing pairwise rank correlations between ensemble members for TXx, maximum temperature in the Mar-May season.
(A) USA region, (B) China region. Leadtime 2 for the USA region has a median correlation is greater than 0.25, and therefore that lead time was excluded from the analysis. Grey shading shows the confidence intervals of the boxplot statistics (whiskers: 1.5x interquartile range, box limits: interquartile range and centre line: median), based on a permutation test with 5% significance level (see Kelder et al. 2020).

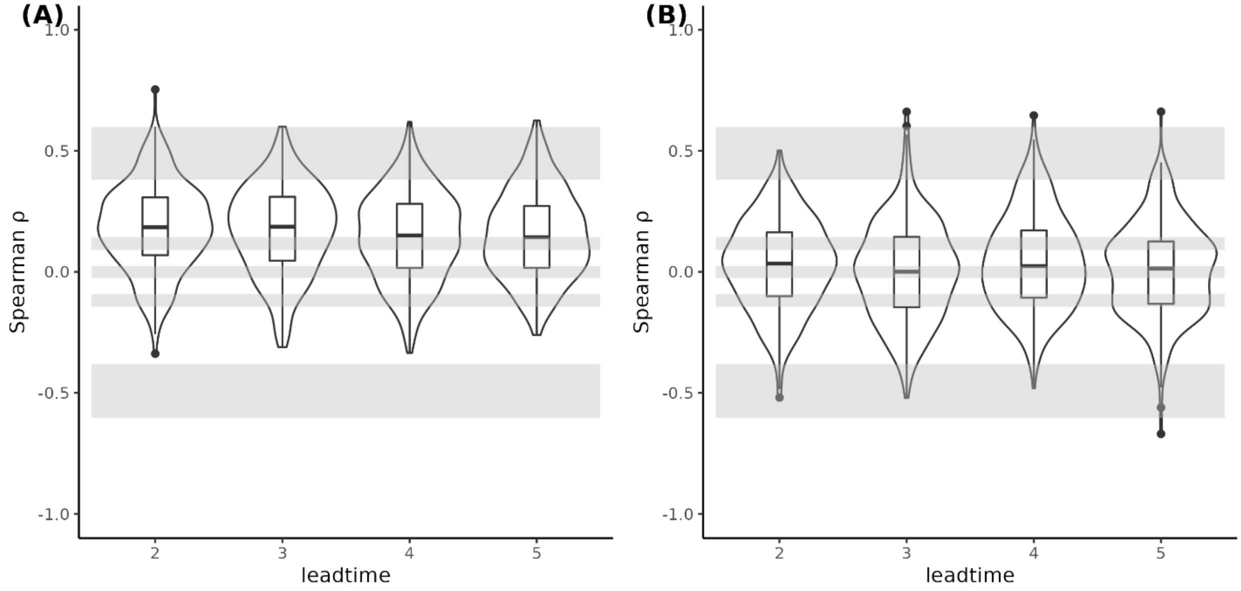

Supplementary Figure 2. As in SI1 for total precipitation in Mar-May. No leadtimes were excluded.

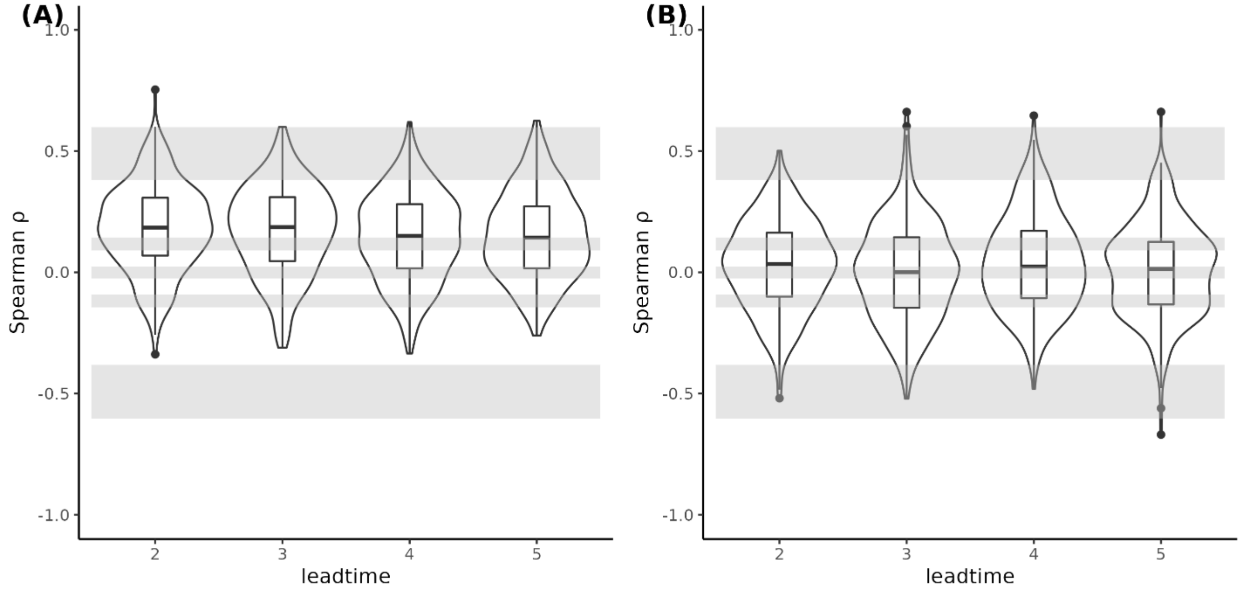

Supplementary Figure 3. Stability across lead times for TXx, showing the climate variable plotted by lead time, to verify that none of the lead times show drift. (A) USA region, (B) China region

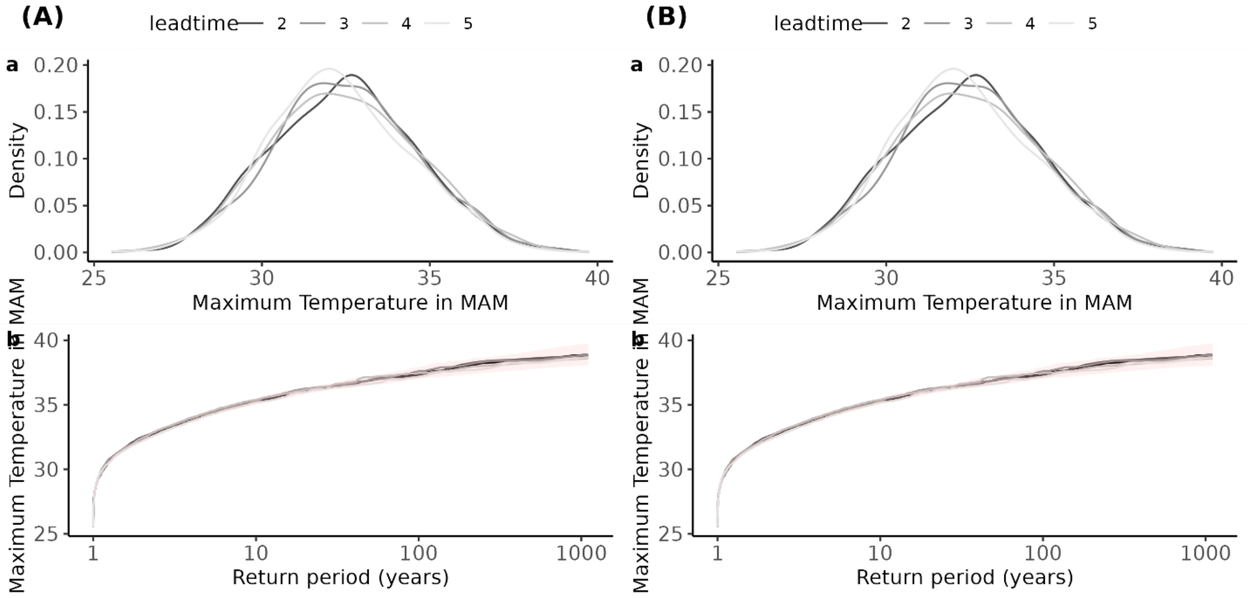

Supplementary Figure 4. As in SI2 for total precipitation in Mar-May.

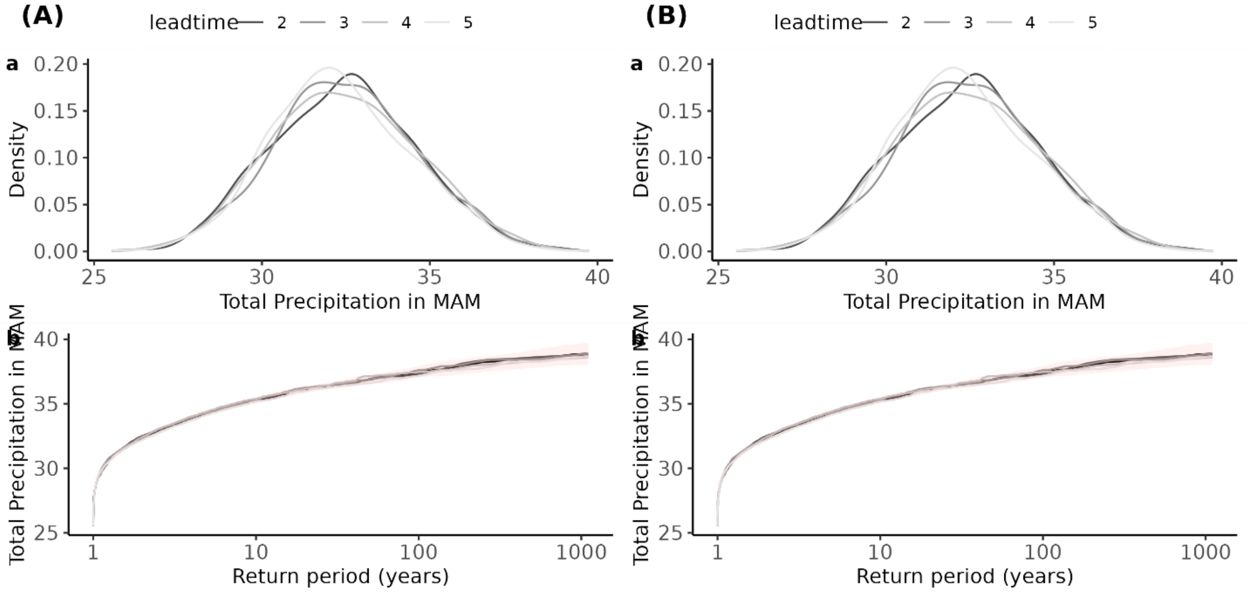

Supplementary Figure 5. Fidelity of the ensemble to observations for TXx in USA region in terms of the mean, standard deviation, skewness, and kurtosis.

The pdf of UNSEEN results is plotted in grey, with the 95th percentile of the ensemble indicated by black dotted lines. The observed value is plotted as a blue vertical line. We note below the figure whether there was any bias-correction applied.

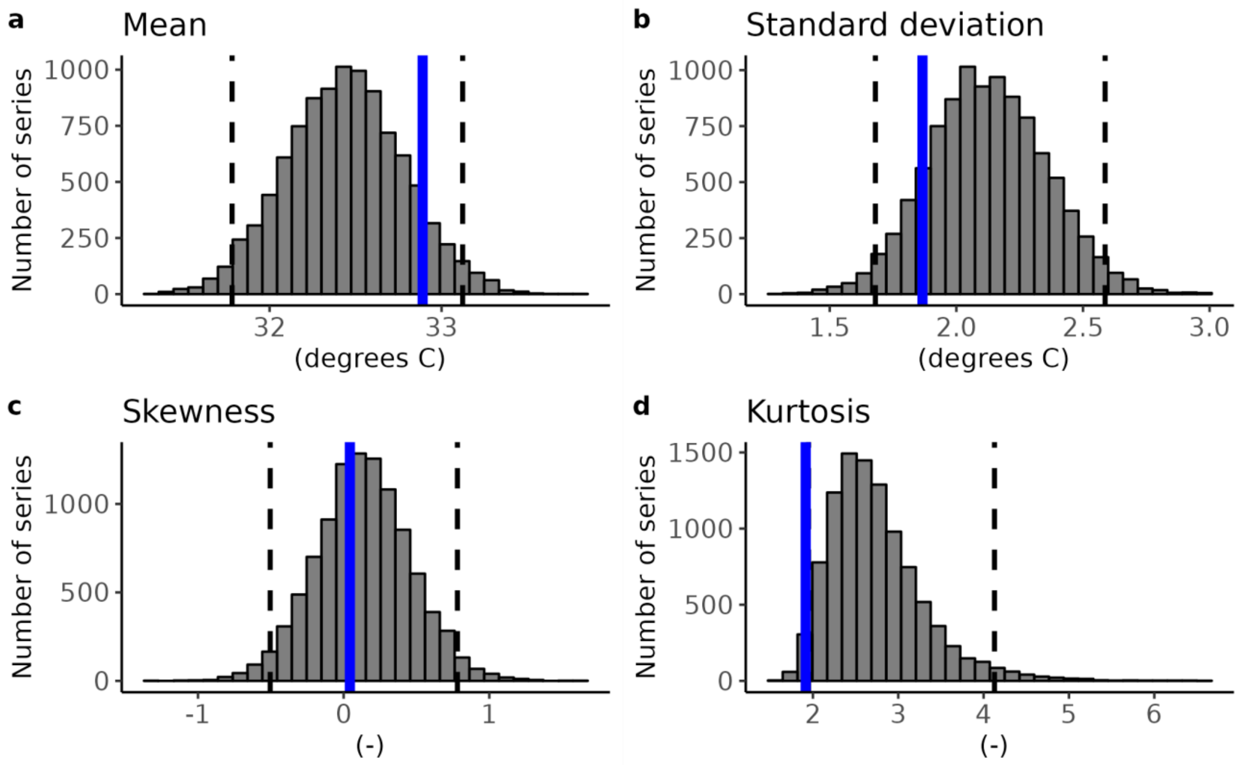

Supplementary Figure 6. As in SI5 for total precipitation, USA region in Mar-May.

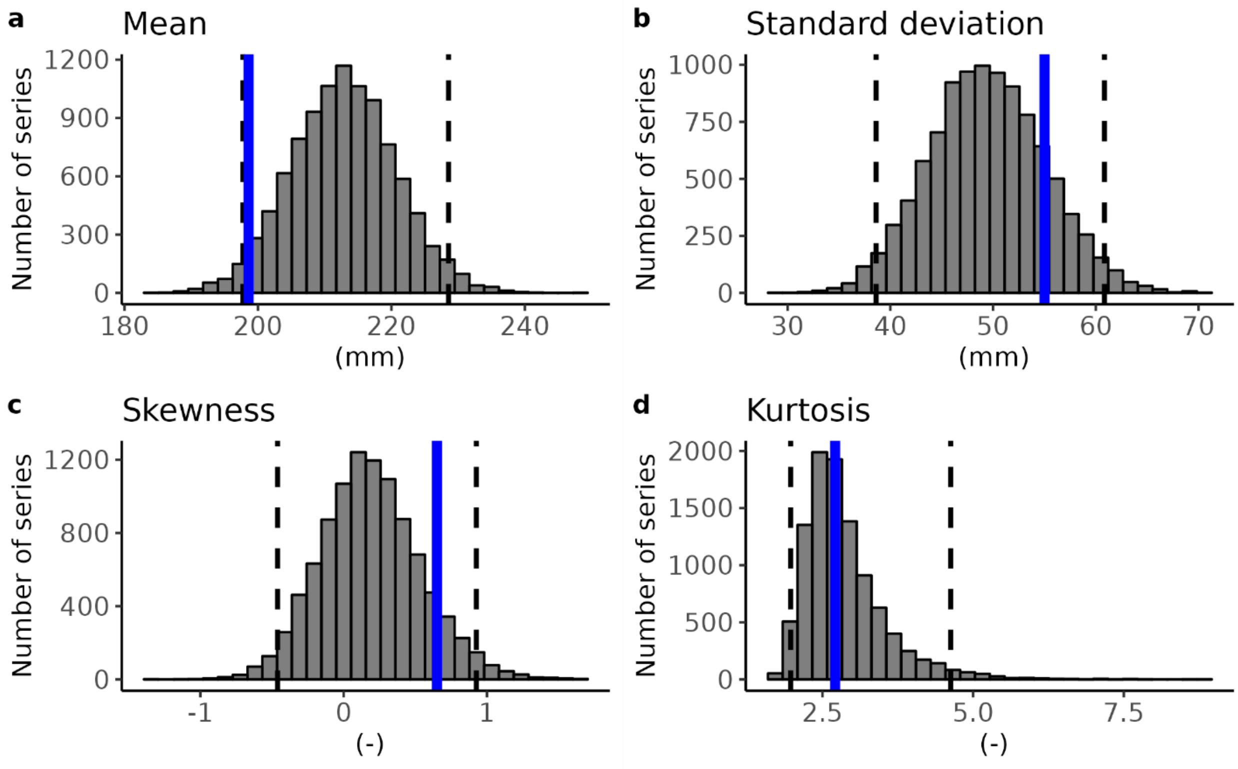

Supplementary Figure 7. As in SI5 for TXx in Mar-May, China region.

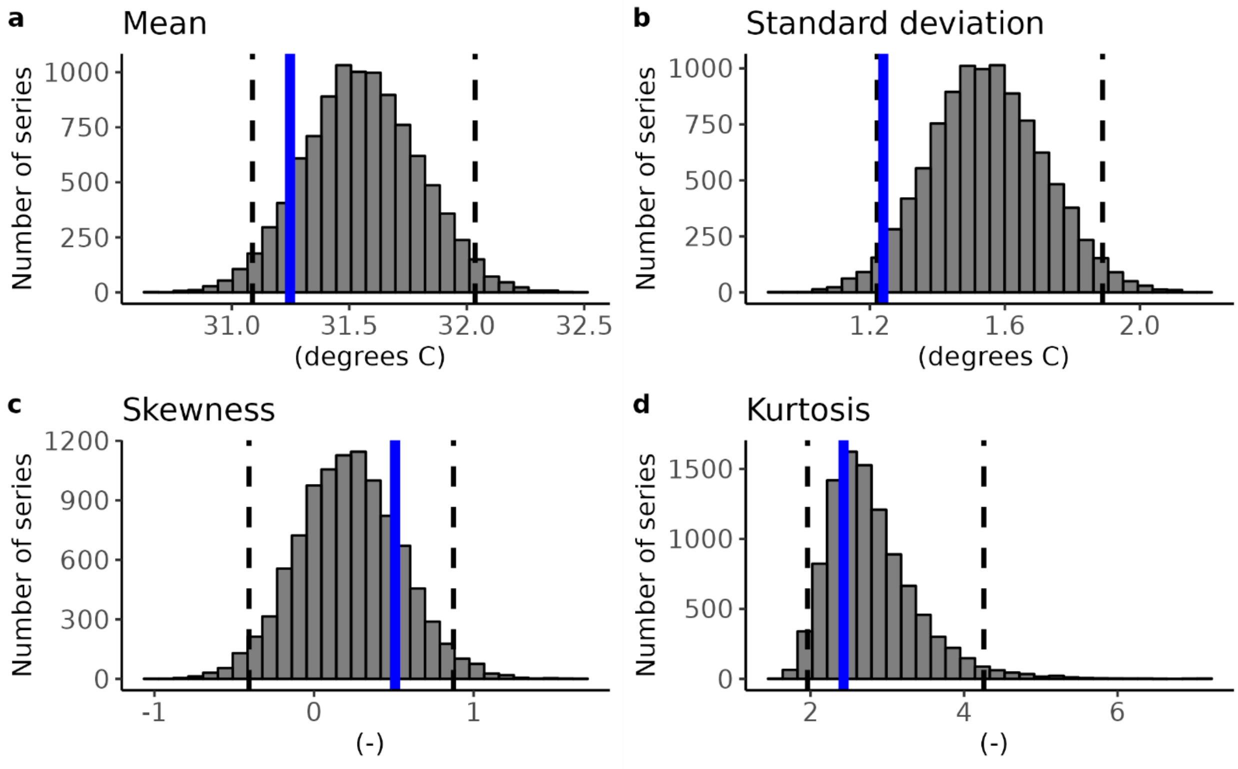

Supplementary Figure 8. As in SI5 for total precipitation in Mar-May, China region.

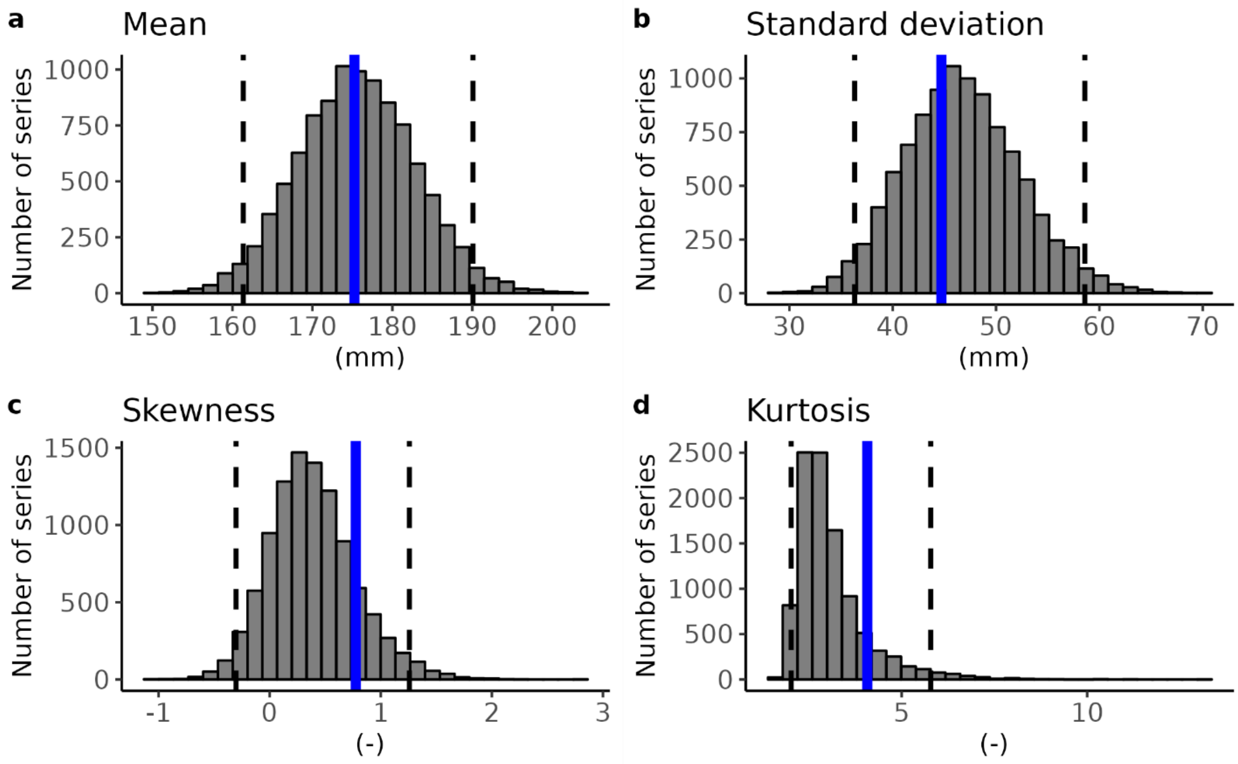

Supplementary Figure 9. Relationship between temperatures in northeastern China and midwest USA study regions in the Mar-May season.

(a) TXx for the Mar-May season in each study area. The linear relationship is plotted in grey, slope of 0.06 (0.03-0.09).

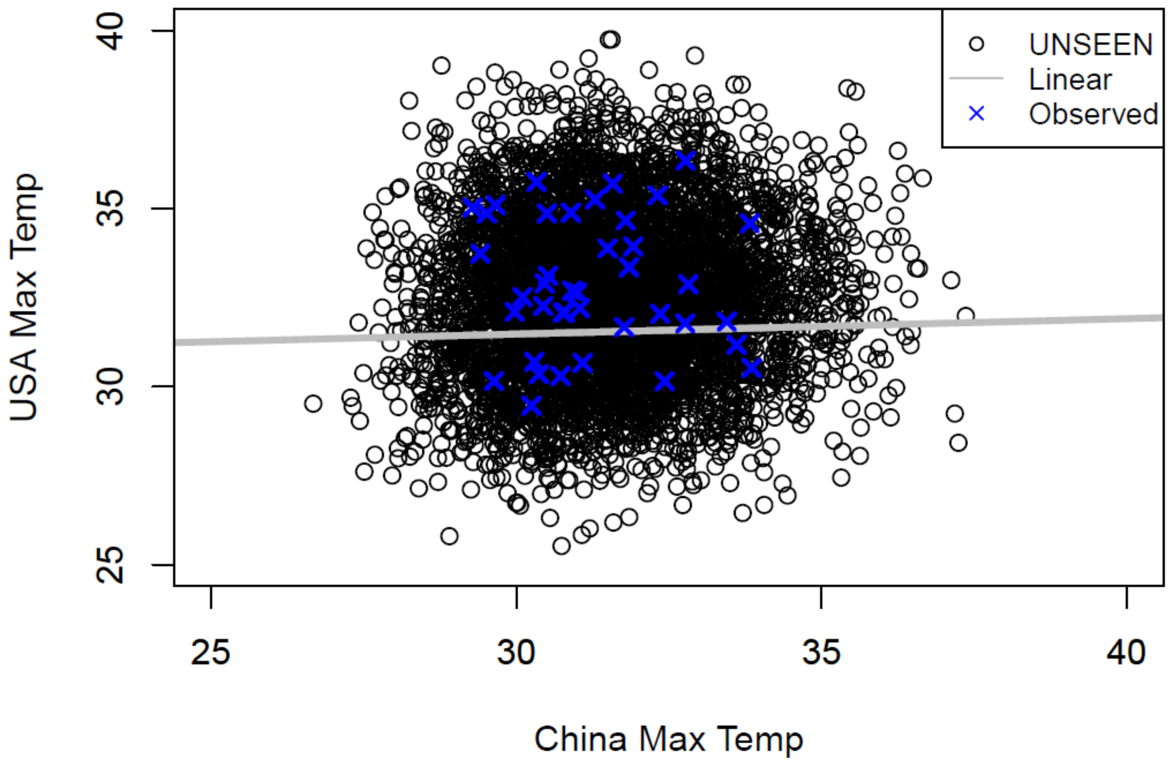

(b) Number of enzyme breakdown days in the Mar-May season in each study area.

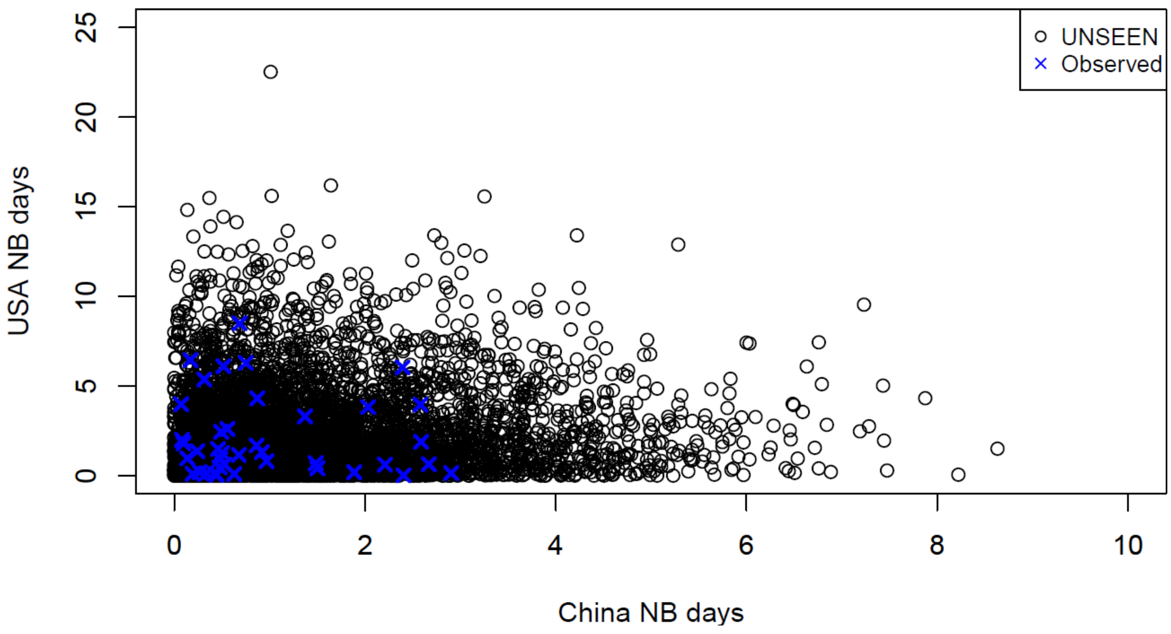

Supplementary Figure 10. Geopotential height and wind anomalies at 500mb associated with the 10 wettest March-May seasons in the USA study area. As in Figure 5 in the main text.

The study area is indicated by a black box in the midwestern USA. Each individual ensemble member is depicted in plots A-J, with the year of the SEAS5 forecast indicated above. Plots may have the same year, but were generated by different ensembles at different lead times.

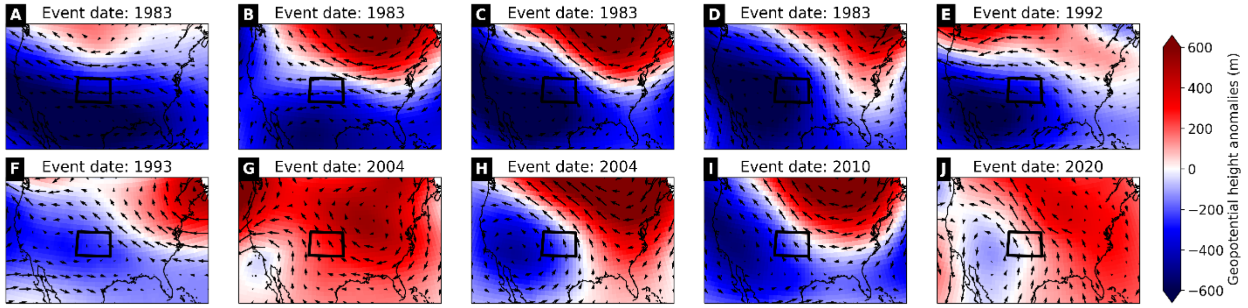

Supplementary Figure 11. As in SI10; geopotential height and wind anomalies at 500mb associated with the 10 driest seasons in the USA study area.

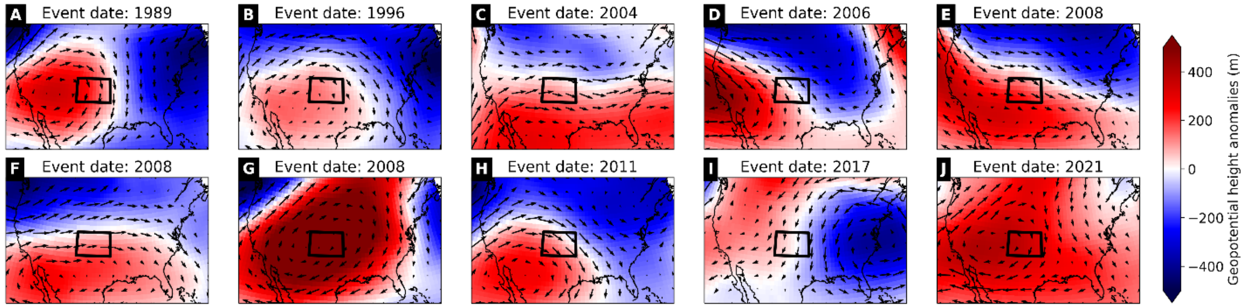

Supplementary Figure 12. As in SI10; geopotential height and wind anomalies at 500mb associated with the 10 seasons of most days above enzyme breakdown threshold in the USA study area.

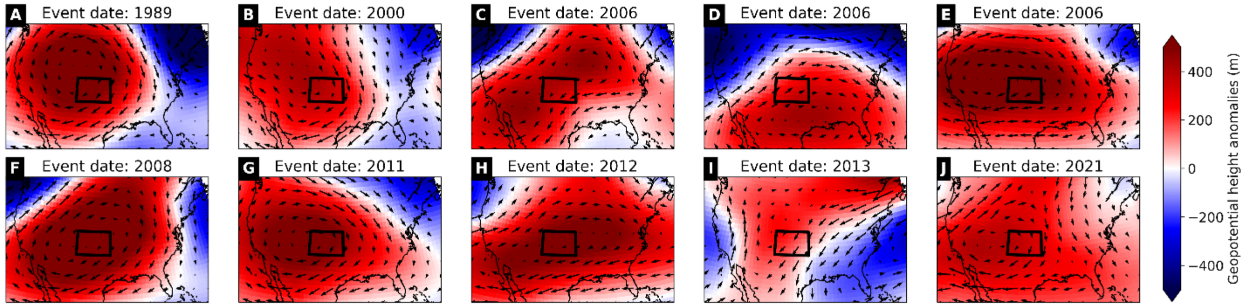

Supplementary Figure 13. As in SI10; geopotential height and wind anomalies at 500mb associated with the 10 wettest March-May seasons in the China study area.

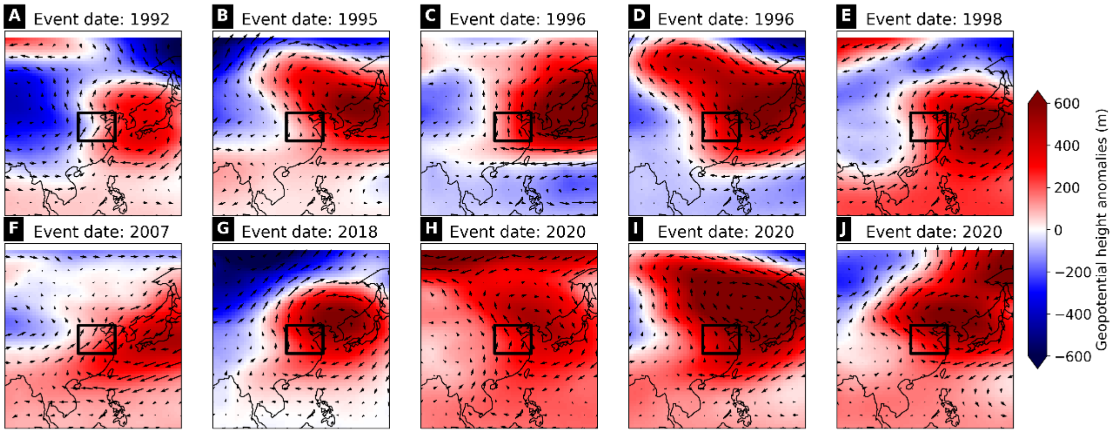

Supplementary Figure 14. As in SI10; geopotential height and wind anomalies at 500mb associated with the 10 driest seasons in the China study area.

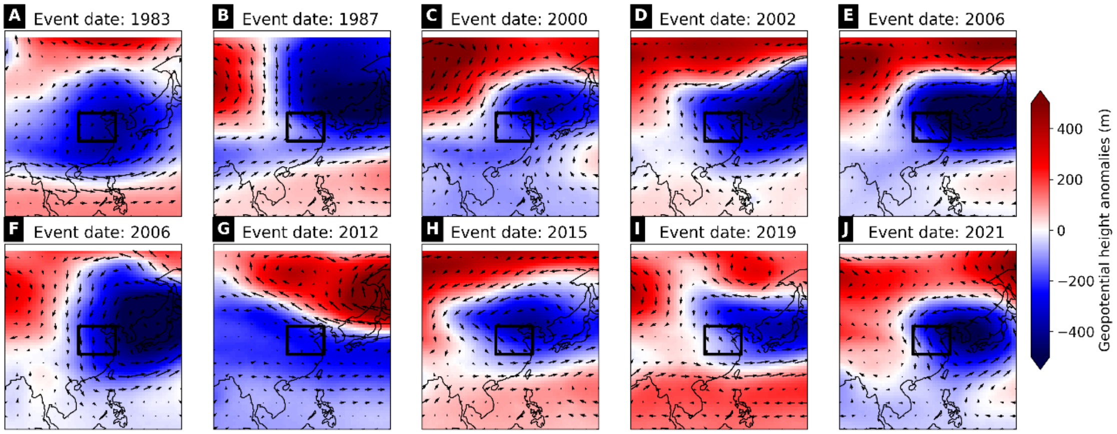

Supplementary Figure 15. As in SI10; geopotential height and wind anomalies at 500mb associated with the 10 seasons of most days above enzyme breakdown threshold in the China study area.

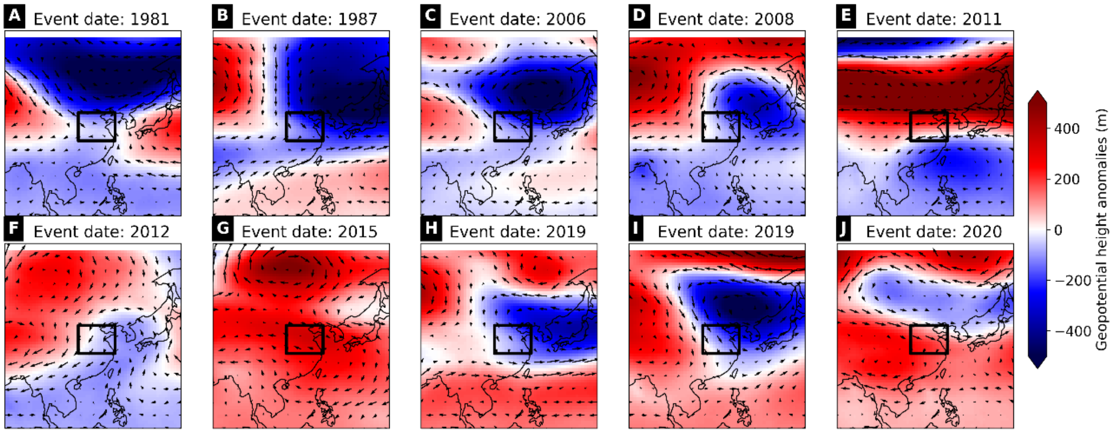

Supplementary Figure 16: Compound events. As in SI10, geopotential height and wind anomalies at 500mb associated with the 10 events that produce the most days above enzyme breakdown threshold in BOTH the USA and China study regions.
These are the 10 events that appear in each region's list of top 250 hot events.

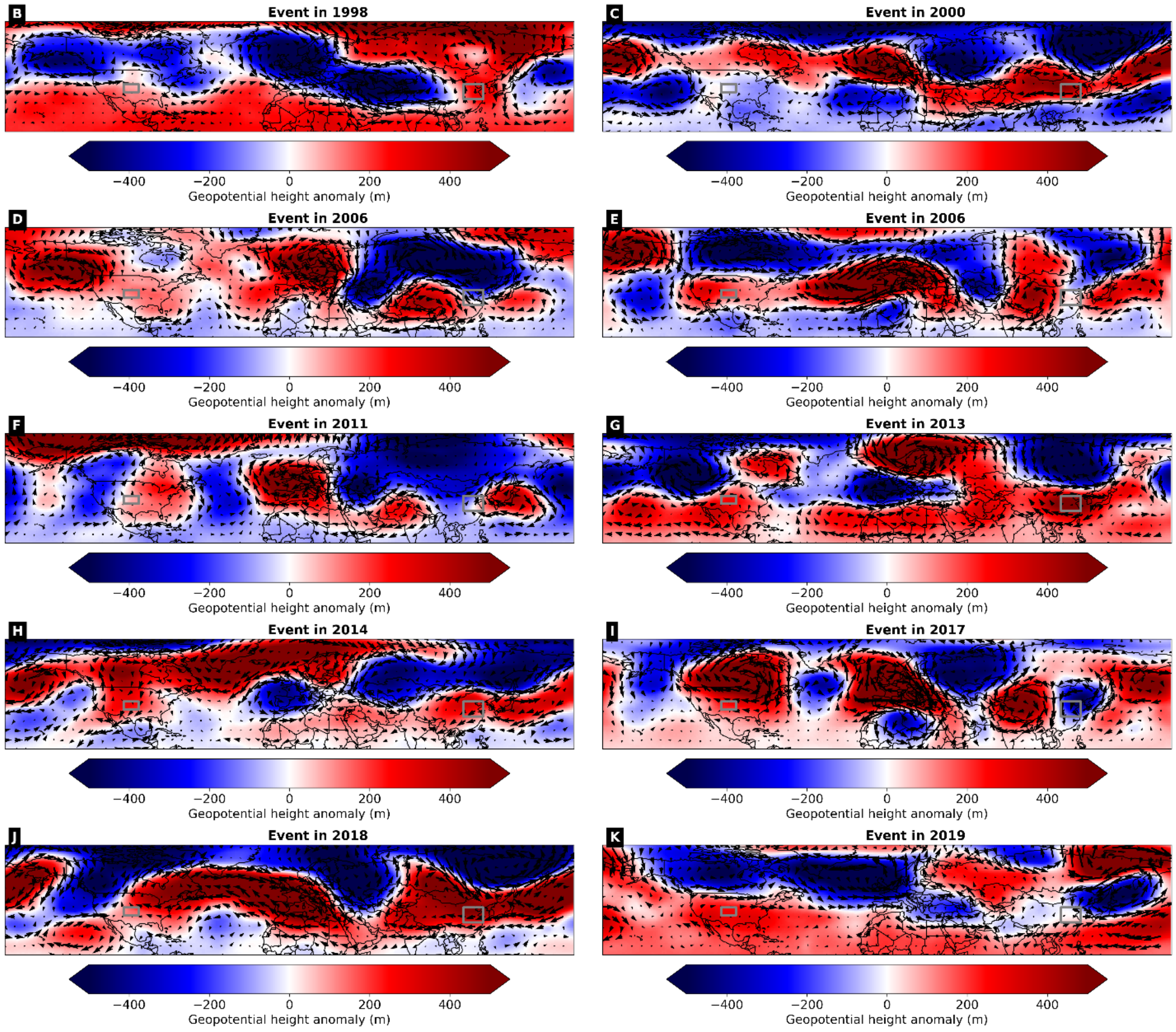

Supplement: Supplementary file 1 — Supplementary Information Coughlan de Perez et al. Wheat UNSEEN USA China [file 41612_2023_361_MOESM1_ESM.pdf]
